# Numerical Modeling of Physical Cell Trapping in Microfluidic Chips

**DOI:** 10.3390/mi14091665

**Published:** 2023-08-26

**Authors:** Sara Cardona, Nima Mostafazadeh, Qiyue Luan, Jian Zhou, Zhangli Peng, Ian Papautsky

**Affiliations:** Department of Biomedical Engineering, University of Illinois, Chicago, IL 60607, USA

**Keywords:** microfluidic cell trapping, hydrodynamic trapping, physical trapping, finite element method, cell isolation

## Abstract

Microfluidic methods have proven to be effective in separation and isolation of cells for a wide range of biomedical applications. Among these methods, physical trapping is a label-free isolation approach that relies on cell size as the selective phenotype to retain target cells on-chip for follow-up analysis and imaging. In silico models have been used to optimize the design of such hydrodynamic traps and to investigate cancer cell transmigration through narrow constrictions. While most studies focus on computational fluid dynamics (CFD) analysis of flow over cells and/or pillar traps, a quantitative analysis of mechanical interaction between cells and trapping units is missing. The existing literature centers on longitudinally extended geometries (e.g., micro-vessels) to understand the biological phenomenon rather than designing an effective cell trap. In this work, we aim to make an experimentally informed prediction of the critical pressure for a cell to pass through a trapping unit as a function of cell morphology and trapping unit geometry. Our findings show that a hyperelastic material model accurately captures the stress-related softening behavior observed in cancer cells passing through micro-constrictions. These findings are used to develop a model capable of predicting and extrapolating critical pressure values. The validity of the model is assessed with experimental data. Regression analysis is used to derive a mathematical framework for critical pressure. Coupled with CFD analysis, one can use this formulation to design efficient microfluidic devices for cell trapping and potentially perform downstream analysis of trapped cells.

## 1. Introduction

Microfluidic trapping of cells has emerged as a promising tool for advancing medical and biological research by enabling investigations into cell-to-cell interactions and signaling [1,2], immunotherapies [3], drug responses [4], and stem cell differentiation [5]. Passive cell trapping using physical obstacles, such as post arrays, is a commonly employed microfluidic strategy. In this approach, cells are hydrodynamically trapped by blocking the flow of cells larger than the gap between adjacent posts. The size of the post spacing must be adjusted in proportion to the target cell size, with the gap typically designed to be approximately 25% of the captured cell size [6]. The shape, dimensions, and layout of these posts have been shown to improve capture efficiency, reduce clogging, and reduce cellular shear stress [7].

Arrays of polygonal microposts, including triangular and diamond shapes, have been found to be effective in size-based trapping as cells and cell clusters can be held at vertex angles. Sarioglu et al. [8] reported the use of triangular posts for label-free capture of circulating tumor cell (CTC) clusters from whole blood, while Gao et al. [9] applied such posts to block CTCs and white blood cells. Trapping with diamond microposts is also an efficient size-based trapping method, as the diamond post shape reduces the gap length between any two pillars and decreases pressure at the filter region, thus improving trapping efficiency and cell viability [7]. Zhu et al. [10] optimized the trapping array with diamond-shaped pillars in a zigzag layout, while Zhou et al. [11] designed a diamond-shaped post array to capture CTCs from blood.

Cell trapping at high efficiency is particularly critical for CTCs and their clusters due to rarity. CTCs are known to disseminate to distant sites in the body through the bloodstream as part of the cancer metastatic process and are often found in extremely low numbers, with fewer than 100 CTCs or 10 clusters per 10 million leukocytes and 5 billion erythrocytes in 1 mL of whole blood [12]. While immunoselection [13,14], magnetophoresis [15,16,17,18,19], or dielectrophoresis [20,21] can be used to isolate these rare cells directly from blood, microfluidic size-based physical cell traps have emerged as a popular alternative.

When designing microfluidic devices for cell trapping, the critical pressure is an important parameter to optimize, as it determines the effective pressure required to capture the target cells while avoiding the capture of unwanted cells [22]. It is influenced by various factors such as cell size, cell shape, pillar spacing, and pillar geometry. By optimizing these design variables, it is possible to improve the efficiency and selectivity of cell trapping and minimize the loss of target cells during downstream analysis. However, experimental determination of these design parameters is challenging and time-consuming. Numerical simulations can be used in conjunction with experimental analysis to better optimize the geometric parameters of the device [23]. While there are examples of trapping device optimization using numerical methods, these are mainly computational fluid dynamic (CFD) studies that focus on particle-fluid dynamics rather than particle-pillar interactions [24,25,26,27,28,29]. As a result, these works lack a comprehensive mechanical analysis.

Optimization of microfluidic trapping devices is crucial for the effective isolation of CTCs, as they are extremely rare. Various models have been developed to describe the behavior of cancer cells as they pass through micro-constrictions. Previous work [30] obtained consistent results by describing a cell as a spring-connected network whose interaction with the fluid is implemented using Immersed Boundary Method (IBM). Balogh et al. [31] proposed the membrane model, a hybrid approach that enables the representation of cells as solid membranes enclosing a viscous fluid. The solid membranes provide a stable and rigid framework that can maintain its shape, while the viscous fluid enclosed within the membrane can flow and change shape over time. However, these models may not be applicable for optimizing the geometry of trapping devices that use physical obstacles to capture CTCs since the concept of trapping is opposite to that of passage through a microcapillary. Therefore, a different approach is needed to optimize the geometry of trapping devices and determine the critical pressure for trapping cancer cells.

In this work, we propose an in silico model that can be used to analyze the behavior of cancer cells as they come into contact with pillars in a microfluidic trapping unit. By numerically simulating this process, we aim to provide a robust estimation of the critical trapping pressure with respect to changes in cell morphology and trapping unit geometry (i.e., post spacing). This can help set the working conditions of the device to ensure that the critical pressure of blood cells is exceeded while the critical pressure of the target CTCs is not, thus enhancing the efficiency and size selectivity of CTC isolation. In summary, the key novelty of this work includes a purely mechanical analysis of cell trapping in microposts of reduced geometry, derivation of a simple and robust model to estimate the effective trapping pressure for capturing CTCs inside microposts, and integration of the model across various cell types and geometries to demonstrate its generalizability beyond the scenario experimentally evaluated in this study.

## 2. Experimental and Numerical Methods

### 2.1. Device Fabrication

**Figure 1:**
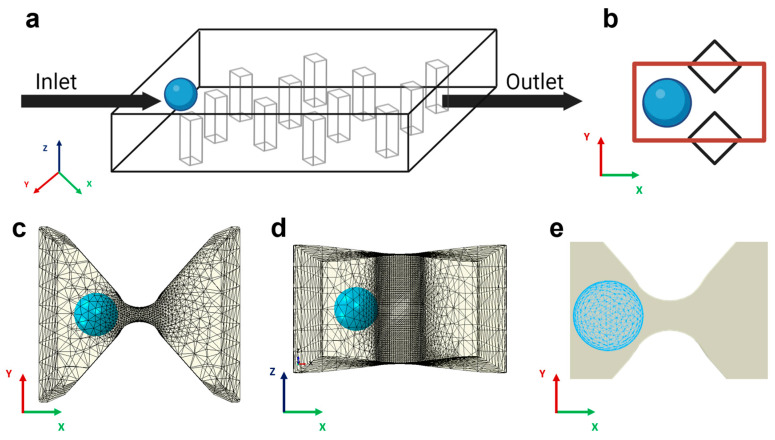
Model schematics and FEM discretization. (**a**) Sketch of the trapping device with diamond pillars interposed between the inlet, the outlet, and the cancer cells (colored light blue). For illustrative purposes, a homogenous spatial distribution of pillars is shown. (**b**) Top view 2D representation of the computational domain as the space between two adjacent posts (marked in red). (**c**) The same area is represented in the simulation. The gap region, modeled as a discrete rigid body, is meshed with linear triangular elements. The computational cost of the simulation is reduced by using the local node definition: a finer mesh is adopted in the region closest to the capture area, and a coarser mesh is selected in the non-contact regions. (**d**) Lateral view of the 3D trapping unit. (**e**) Cancer cells meshed with quadratic tetrahedral elements.

The microfluidic device contained an array of diamond posts of 70 µm × 70 µm (diagonals) × 50 µm (height) with decreasing gap size from the inlet to the outlet (Figure 1a). The post array was organized into four columns: the 1st column (closest to the inlet), with gap size progressively decreasing from 50 to 14 µm; the 2nd array, with a gap size of 10 µm; the 3rd array, with a gap size of 8 µm, and the 4th array (closest to the outlet) with a gap size of 4 µm. The 50 µm high microchannel was fabricated in polydimethylsiloxane (PDMS) using dry film photoresist masters, as we described previously [32]. Briefly, dry films (ADEX 50, DJ MicroLaminates Inc., Sudbury, MA, USA) were used to pattern the microchannels on 3″ silicon wafers. The microchannels were then replicated in PDMS (Sylgard 184, Dow Corning^®^, Midland, MI, USA) and bonded to 1″ × 3″ glass slides (Fisher Scientific, Hampton, NH, USA) to form sealed devices after oxygen surface plasma treatment (PE-50, Plasma Etch Inc., Carson City, NV, USA) for 20 s. Inlet and outlet ports were manually cored using a biopsy punch with an outer diameter of 1.5 mm (TedPella Inc., Redding, CA, USA).

### 2.2. Sample Preparation

The non-small-cell lung cancer (NSCLC) cell line A549 was purchased from ATCC (Manassas, VA, USA) and maintained as specified. The cell line was incubated in RPMI 1640 medium supplemented with 10% (*v*/*v*) fetal bovine serum (FBS) and 1% (*v*/*v*) 100× antibiotic-antimycotic solution. Cells were maintained at 37 °C and 5% CO_2_. For trapping experiments, cells at 70–80% confluency were treated with 0.25% trypsin-EDTA, centrifuged at 1000 rpm for 5 min, and re-suspended in 2 mL of PBS with a final concentration of 50,000 cells/mL. All cell culture supplies were purchased from Fisher Scientific (Waltham, MA, USA). Fetal bovine serum (FBS) was purchased from GeminiBio Inc. (West Sacramento, CA, USA).

### 2.3. Cell Trapping Experiments

Cell sample solution (50,000 cells/mL in PBS) was loaded in a syringe (Norm-Ject^®^, Air-Tite Inc., Jonesboro, AR, USA), which was connected to 1/16″ Tygon^®^ tubing (Cole-Parmer, Vernon Hills, IL, USA) using proper fittings (IDEX Health and Science LLC, New York, NY, USA). The other end of the tubing was secured to the device inlet. To avoid cell loss, both the connecting tubing and the syringe were treated overnight with an anti-adherence solution. A syringe pump (Legato 200, KD Scientific Inc., Holliston, MA, USA) was used to sustain a stable flow rate of 10 µL/min.

### 2.4. Experimental Data Acquisition and Analysis

The microchannel was placed on the stage of an inverted microscope (IX83, Olympus America, Center Valley, PA, USA). Bright-field images were acquired using a high-resolution sCMOS camera (Andor Zyla 5.5, Oxford Instruments, Santa Barbara, CA, USA) mounted on the microscope along with the cellSense imaging software (v4.1, Olympus America, Center Valley, PA, USA). At least 50 images were acquired with magnification 20× to 60×. All images were analyzed using ImageJ^®^ (v1.53t, NIH, Bethesda, MD, USA) to enumerate trapped cells and to confirm gap dimensions. The gap efficiency was computed as the fraction of occupied traps for each gap size.

### 2.5. Computational Domain

To model the mechanics of physical trapping in a microfluidic device, we conducted finite element analysis using Abaqus/Explicit (v2020, Dassault Systemes Simulia, Inc., Johnston, RI, USA). Because the physical trapping process involves contact between deformable cells and the trapping unit and cell deformation can be quite large, the finite element method (FEM) with explicit time integration (e.g., Abaqus/Explicit) is preferred over FEM with implicit time integration (e.g., Abaqus/Standard). The simulation domain consisted of a single 3D trapping unit formed by two adjacent diamond-shaped pillars with gap distance varying from 4 µm to 10 µm (Figure 1b). We modeled the trapping unit as a rigid body because the relatively higher stiffness of PDMS (~1.7 MPa) compared to that of cells (350–650 Pa) leads to a significant reduction in computational cost. The motion of the rigid constraint was determined solely by the reference node rigidly fixed at the center of the gap, and the rigid shape remained static throughout the simulation. For the domain mesh, extra fine rigid triangular elements (R3D3) were employed on the contact region to guarantee accurate results, while a coarser mesh was employed around to reduce the computational effort (Figure 1c,d).

### 2.6. Computational Model of Cancer Cells and Loading Conditions

Cancer cells were modeled as 3D deformable spherical bodies with a diameter ranging from 10 to 30 µm [33]. We considered the cell as a homogeneous elastic body and neglected the detailed internal structures, such as the nucleus, because we will use the effective moduli (such as Young’s modulus) measured experimentally, which reflects the overall mechanical properties of the cell (including the nucleus) rather than local stiffness. Further, our main purpose in this study is to estimate the critical pressure required to push the cell through rather than the detailed stress distribution in the cell or its surface. Based on our experimental observations and evidence from the literature [34], we employed a neo-Hookean hyperelastic model to describe the material constitutive behavior of the cell. Young’s modulus of the cells we used (A549 cells) was measured in a range of 350 Pa to 650 Pa [35,36], reflecting the higher plasticity and invasiveness that malignant tumor exhibits during metastatic spreading. Poisson’s ratio was set to 0.3. Designed to be used in complex contact simulations, modified quadratic tetrahedral elements (C3D10M) were adopted to finely mesh the single cell (Figure 1e).

To accurately estimate the hydrodynamic pressure between pillars, a CFD analysis was conducted in COMSOL Multiphysics (v5.6, COMSOL Inc., Burlington, MA, USA). Based on the assumption of a laminar regime, the incompressible Navier–Stokes equations were solved under the steady state condition inside a computational domain representing the trapping device. The flow parameters were obtained by solving the conservation of momentum and mass, which can be articulated as follows:

(1) $$\rho \left(u\cdot \nabla \right)u=\nabla \cdot \left(-pI+\mu_{s}\left(L+L^{T}\right)\right)$$

(2) ∇·u=0

where ρ, $\mu_{s}$, and ***I*** are the fluid density, solvent viscosity, and identity matrix, respectively. ***L*** is the velocity gradient tensor. Fluid density and solvent viscosity were set to 1000 $\mathrm{kg}/m^{3}$ and 1 mPa·s, respectively. The fluid velocity was prescribed at the inlet based on the flow rate of the microfluidic syringe pump, while zero pressure was prescribed at the outlet. The walls were ascribed with no-slip boundary conditions. The pressure drop along the arc length of a path going through a gap was estimated in the context of 6 μm gap and flow rates of 1, 5, and 10 μL/min.

To convey the fluid dynamic results into the mechanical simulation, the cell was partitioned into two regions, and a parabolic pressure was applied in the front region to mimic the flow pressure in the trapping zone. The analytical expression field for the pressure acting in the x direction was defined as follows:

(3) $$f\left(y,z\right)=1-\left(\frac{y^{2}}{r^{2}}+\frac{z^{2}}{r^{2}}\right)$$

where *r* is the cell radius. The contact between deformable cells and the rigid PDMS wall surfaces was modeled as a contact frictionless problem acting at the interface, with general contact constraining nodes of one surface from penetrating the other. A hard pressure overclosure was adopted to minimize surface penetration at gap locations.

### 2.7. Numerical Data Analysis and Critical Pressure Evaluation

To determine the critical pressure relationship with the input variables, a nonlinear regression analysis was conducted in Matlab (R2021b, Mathworks Inc., Natick, MA, USA). This analysis allowed us to identify the functional form of the relationship between pressure and the input variables and to estimate the values of the parameters in the relationship. To assess the predictive capability of the model, new data were generated, and the simulated pressure was compared with the estimated pressure. The mathematical model was validated experimentally by using results reported in the literature [31], and the accuracy and precision of the model were assessed through statistical analysis.

## 3. Results and Discussion

### 3.1. Cell Trapping in Microfluidic Device

To evaluate trapping performance, it was important to assess the size of the A549 cells that we used in this work. A549 is a non-small-cell lung cancer (NSCLC) cell line that has been used to mimic lung cancer CTCs in prior studies [37,38,39]. A mean diameter of 17.27 ± 2.91 μm was measured for A549 cells, although others have reported smaller values closer to 15 μm [40]. The range of the measured cells was quite broad, from about 12 µm to nearly 28 µm. Based on the previously described criteria of using 25% of the captured cell size [6], to capture A549 cells, the gap between trapping posts must be approximately 3 μm for the smallest cells in the range and approximately 7 μm for the largest cells. The trapping array spacing of >10 μm should not yield any trapped cells.

**Figure 2:**
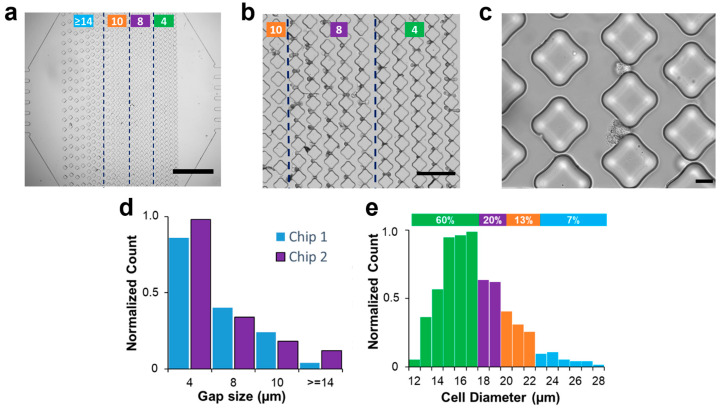
Physical trapping of A549 cells in microfluidics. (**a**) Bright-field image of the trapping device with vertical arrays of diamond micro-posts (scale bar: 1 mm). Based on the column spacing, the device comprises 4 different regions (marked with the green lines). The leftmost has a gap size greater than 14 µm and serves as a filtering device for cell aggregates. The two middle regions are designed with gap sizes of 10 µm and 8 µm to trap single cells. The arrays closer to the outlet are characterized by tiny spaces aimed at retaining highly deformable cells. (**b**) Bright-field image of A549 cells trapped in the arrays closer to the outlet (scale bar: 200 µm) with column spacing between 10 (leftmost arrays) and 4 µm (rightmost arrays). (**c**) A zoomed image of effective trapping in 8 µm gap size (scale bar: 20 µm). (**d**) Columns chart representing the normalized number of single cancer cells captured in two different chips for each column spacing. (**e**) Diameter distribution of A549 cancer cells. The histogram shows a correspondence between the diameter distribution of cells and their trapping distribution, with smaller cells being associated with trapping in smaller gaps closer to the outlet, while larger cells were trapped in the initial arrays with larger gap sizes. Green, purple, orange, and blue colors represent gap spacing of 4, 8, 10, and >14 µm, respectively.

The microfluidic chip was designed to trap cells using diamond pillars that allow cells to maintain their integrity and healthy state, as reported from viability studies by Zhou et al. [11]. From a mechanical standpoint, the diamond shape provides several benefits. While the frontal cellular interaction with micro-posts remains constant when using triangular-shaped posts, the lateral interaction is improved in symmetric diamond-shaped obstacles due to a consistent reduction in the shear stresses. In addition, compared to the triangular profile, the exposed surface is larger in diamond obstacles, implying that the physical trapping method can be enhanced with surface-treated approaches. The diamond post array in the microfluidic chip gradually reduced the gap size between posts from the inlet towards the outlet to allow a step-by-step analysis of the trapping process. This design is illustrated in Figure 2a. Arrays of trapping posts of various spacing were placed inside a broad microfluidic chamber. The larger gap spacing was used near the inlet for the capture of larger cells, while smaller cells were expected to traverse the trapping array toward the smaller trapping spacing near the outlet. The four specific gap sizes in the post array were >14 µm, 10 µm, 8 µm, and 4 µm. Figure 2b illustrates a representative bright field image of A549 cells trapped in the arrays with 8 µm and 4 µm gap spacing. A further close-up image in Figure 2c shows individual cells trapped between posts with an 8 µm gap. Thus, as anticipated, the microfluidic chip was capable of trapping A549 cells.

The uneven disposition of posts created a polarization in the distribution of captured cells. When a constant flow rate of 10 µL/min was imposed, the A549 cells easily passed through gaps > 14 µm, with only 5% trapped in this first array. The smaller gap sizes of 10 µm, 8 µm, and 4 µm resulted in trapping of 13%, 23%, and 59%, respectively. Similar results were obtained over multiple experiments in two microfluidic chips (Figure 2d). The results in Figure 2e suggest a correlation between the two distributions, whereby smaller cells with diameters ranging from 12 to 17 μm constituted the majority (60%) of the population, which were found to be associated with trapping in smaller gaps located closer to the outlet. Likewise, cells with diameters of 18 and 19 μm formed 23% of the population and were found to be associated with gap sizes of 8 μm, while cells with diameters of 20 to 22 μm, forming 13% of the population, were associated with gap sizes of 10 μm. Notably, cells with diameters larger than 23 μm constituted 5% of the population and were trapped in the initial arrays with larger gap sizes. These findings reaffirm the notion that cell size, in conjunction with compliance of cell walls, has a substantial impact on the ability to hydrodynamically trap them. These findings also suggest that the proposed capture criterion of 25% of cell diameter [6] works for the smaller cells, while the larger cell population does not seem to follow this rule. Next, we will apply numerical simulations to investigate the underlying physics behind the trapping process and propose a way to predict critical pressure values based on cell morphology and post-spacing.

### 3.2. Computational Model of Physical Trapping

**Figure 3:**
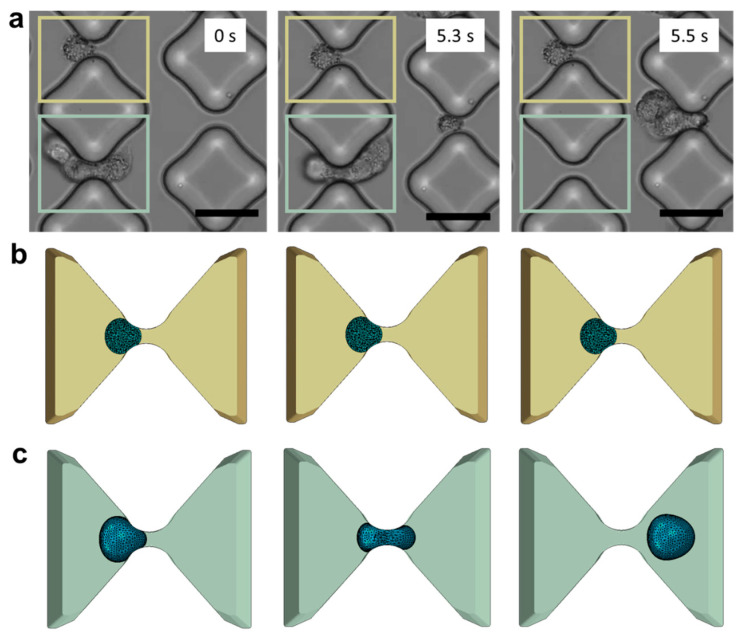
In vitro experiments vs. in silico models. (**a**) De-trapping progression of A549 cancer cells in the testing device (scale bar: 50 μm). Three consecutive frames capture the same device region and the same inter-pillars gap to illustrate a case of successful trapping (marked in yellow) and a case of de-trapping (marked in light green). (**b**) The corresponding phases trapping phases are illustrated in the numerical framework. (**c**) Cell deformability and tendency to elude the obstacles are well captured in the model.

The computational domain was established by selecting the space between two adjacent diamond posts and precisely reproducing the smoothness of their PDMS surfaces. Accurate characterization of the material is crucial to capture the cellular response during collisions with the posts. Cancer cells exhibit greater susceptibility to deformation than healthy cells and tend to undergo softening as they navigate through confined spaces [26,41,42]. A linear elastic model of tumor cells fails to account for this behavior. Despite the limitations of the viscoelastic model in describing the trapping process, various techniques such as micropipette aspiration [43], optical tweezers [44,45], atomic force microscopy [46], and magnetic tweezing cytometry [47] have been developed to assess the viscoelastic properties of cells in recent decades. However, different measuring techniques lead to significant variability in the viscoelastic data, which also varies considerably from cell to cell [48]. Experimental data suggest that metastatic cells undergo stress-induced softening as they navigate through narrow gaps (Figure 3a). We tested various material models and found that the neo-Hookean hyperelastic model is not only simple and robust but also able to capture the experimental observations (Figure 3b,c).

Another important consideration was the selection of the Poisson’s ratio, which is known to vary across biological systems from the cell membrane to the nucleus. Based on experimental measurements reported by others [49], a Poisson’s ratio of 0.3 was selected as it provided the most accurate description of the cell’s peripheral properties in contact with the pillars. This choice was deemed crucial in accurately modeling the mechanical response of the cell during hydrodynamic trapping.

**Figure 4:**
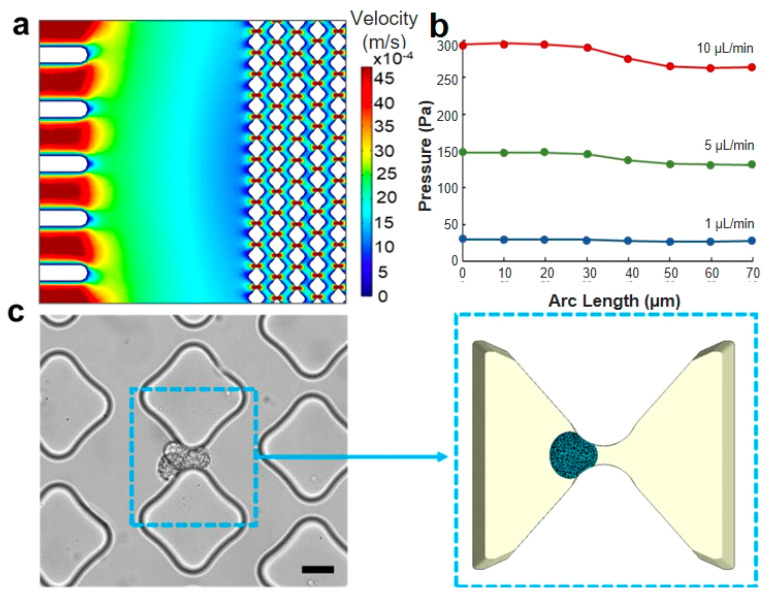
Loading conditions and region of interest. (**a**) CFD simulation of the velocity distribution in the microfluidic trapping device, illustrating multiple traps. (**b**) CFD simulation of the pressure drop along the flow direction in a single trapping unit with a 6 μm gap across flow rates of 1, 5, and 10 μL/min. (**c**) The trapping unit area is depicted in an optical image of the experimental device (scale bar: 20 μm), and the same area is represented in the computational model reported on the right side. The simulation accurately replicates the smoothness of the pillar’s shape and the cell deformability when interfacing with the constraint.

During the trapping experiment, the cell suspension flowed from the inlet toward the outlet of the microfluidic device and would have passed undisturbed if not for the presence of the obstacles. Our analysis focused on studying the mechanical contact between the cancer cells and pillars. However, to gain a comprehensive understanding of the local dynamics, it was necessary to consider the fluid as well. Rather than directly modeling the fluid flow with the cell together, an indirect approach with two sequential steps was taken. First, the fluid velocity and pressure were predicted using CFD simulations in COMSOL. The simulation results indicated that the fluid flow in the proximity of obstacles exhibited a velocity distribution with a parabolic shape (Figure 4a). The pressure magnitude along the arc length of a 6 μm gap is obtained from the CFD simulation (Figure 4b), revealing a negligible longitudinal pressure gradient for the flow rates typically employed in microfluidic trapping experiments. This information was then used to define the pressure distribution applied on the cell membrane in the model, which is a good approximation of the actual pressure experienced by the cell in the actual device (Figure 4c).

### 3.3. Relationship for Estimating Critical Pressure

**Figure 5:**
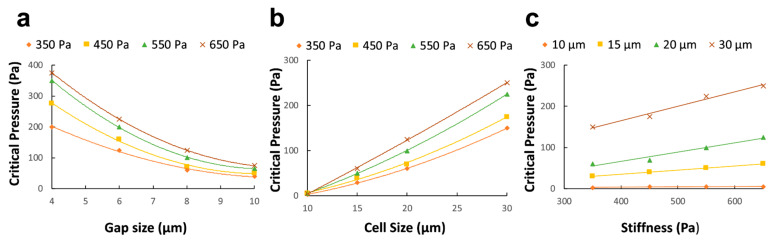
To explicitly define the effect of the optimization parameters on the critical pressure, the critical pressure is plotted against gap size for various cell elasticity when the cell size is kept constant (**a**). Similarly, the gap size is maintained constant, and the corresponding curves are drawn for cell size at varying elasticity (**b**) and cell elasticity at varying cell diameters (**c**).

The approach herein consisted of conducting parallel in vitro and in silico analyses to evaluate the maximum pressure value that enables high throughput while keeping high trapping efficiency. Thus, a range of gap sizes and cell morphologies were evaluated, and a mathematical formula was obtained. A thorough examination of the process from a physical standpoint revealed predominantly a surface-based nature. Thus, the computational model was formulated as a contact problem involving the interaction between a deformable sphere and a rigid body obstructing its free path. To elaborate further, the exposed cellular area and the gap opening can significantly affect the critical pressure. Specifically, larger gaps would provide a more accessible pathway for cells to exit the trapping unit, thus reducing the minimal pressure value required to retain the target in the device. Conversely, thinner spaces create the most suitable condition for increasing the imposed pressure without losing cells (Figure 5a). Moreover, it was observed that the critical pressure is strongly influenced by the size of the target in question. In particular, when the size ratio between the cell and gap dimensions significantly favored the cell, a noticeable rise in critical pressure was needed to enable the cell to escape from the obstacle. Conversely, in situations where the cell size was relatively small, and the ratio between the cell and pillar spacing (i.e., gap size) was reduced, the threshold for critical pressure was expected to decrease markedly (Figure 5b). Lastly, the pressure trend was suspected to be influenced by cellular elastic properties that remained in the linear range during the entire simulation of the contact process (Figure 5c).

The present study used nonlinear regression analysis on numerical simulation data to develop a simple formula of critical pressure based on the geometrical and mechanical parameters characterizing the trapping experiment. Consistent with experimental observations, explicit finite element analysis revealed that successful cell entrapment and escape are significantly affected by the surface contact between the cell membrane and the PDMS pillars. Specifically, the critical pressure (*P_c_*) was found to be quadratically dependent on the ratio of cell diameter (*a*) and gap size (*g*), yielding the following expression:

(4) $$P_{c}=0.027\times E\times {\left(\frac{a}{g}\right)}^{2}$$

where *E* is cell modulus of elasticity. An examination of the computational dataset and the nonlinear regression analysis produced a high *R*^2^ = 0.965 and a low standard error *SE* = 0.44. These statistical metrics indicate a strong correlation between the variables examined, supporting the validity of the empirical model.

To ensure a comprehensive analysis, the relationship between critical pressure and the cell-gap size ratio was further investigated by comparing the quadratic model with linear and cubic models. The results showed that the quadratic model more closely matched the simulated data compared to the linear model (*R*^2^ = 0.798) and the cubic model (*R*^2^ = 0.78). This observation highlighted that when performing hydrodynamic trapping in microfluidic chips, the specific geometry of the trapping unit and the cancer cell dimension must be considered to set the pump operations. To ensure inclusiveness in our simulation study, several test instances were conducted with input parameters not included in the original dataset. Thus, the parameter space was comprehensively explored, and average *SE* = 0.097 and *R*^2^ = 0.9034 confirmed the ability of the model to predict pressure values when cellular morphology and trapping unit geometry significantly vary from the initial dataset. Our findings differ from those of Kojić et al. [50], who identified a linear relationship between critical pressure and cell size and elasticity without considering the impact of the constriction. Through the incorporation of gap size in both our experimental and numerical simulations, we were able to demonstrate that it exerts a significant influence on the critical pressure behavior.

### 3.4. Experimental Validation

**Figure 6:**
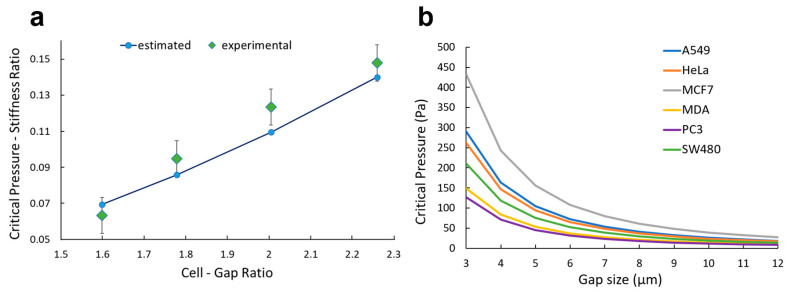
Experimental validation and potential application. (**a**) For gap sizes ranging from 6.8 to 9.8 µm, the critical pressure is plotted both for the experimental data reported by Balogh et al. [31] and ones estimated with our empirical model (Equation (1)). (**b**) Critical pressure is plotted for A549, MCF-7, MDA-MB-231, PC-3, HeLa, and SW-480 based on the morphological values reported in the literature (Table 1).

To assess the reliability of the model in describing the physical trapping process, experimental data found in the literature [31] were used to validate the model. Namely, the critical pressure associated with L1210 cancer cells and constrictions ranging from 6.9 µm up to 9.8 µm were evaluated by adopting our empirical model. Input values were carefully selected to accurately reproduce the experiment under investigation, and the predicted critical pressure behavior was compared with the measured trend. These results are presented in Figure 6a. Our empirical model was found to be highly predictive of the dataset, with regression coefficient *R*^2^ = 0.9095. The statistical significance (*p* < 0.01) indicates that our model accurately captures the relationship between the input parameters and the critical pressure values. Additionally, the Pearson correlation analysis yielded a coefficient *r* = 0.98, confirming the strong correlation between the published experimental results and our model. This validation shows the model to be capable of accurately extrapolating critical pressure values for input parameters outside the initial dataset.

Overall, the combination of strong statistical significance, high correlation coefficient, and effective extrapolation capabilities demonstrated our model to be robust, reliable, and accurate in predicting the critical pressure values to trap tumor cells using the confinement effect (Figure 6a). Nevertheless, our model can be employed to set the working conditions of the syringe pump when on-chip trapping is performed: the user should select an input pressure below the critical value based on the cell type and the design of the device (Figure 6b). For example, A549 and MDA-MB-231 cells, which are of comparable cell size but different stiffness (430 vs. 206 Pa, Table 1), yield similar critical pressure for larger gaps > 8 µm. However, the critical pressure for trapping A549 is nearly 2× higher than for MDA-MB-231 cells when the gap size is reduced to 4 µm. These data can allow improvement in trapping efficiency without limiting the throughput of the device.

## 4. Conclusions

In this work, numerical methods were used to determine a quantitative description of the critical pressure for capturing cancer cells using a microfluidic device with arrays of posts. The mathematical framework was carefully constructed based on the available data, theoretical considerations, and numerical analysis. An existing work [50] introduced a pressure size-stiffness relationship; however, the lack of the gap influence limits the accuracy and versatility of the above in describing the trapping process. Based on our empirical model, the critical pressure is quadratically proportional to the cell-gap size ratio, which means that the distance between pillars strongly affects the entrapment. To our knowledge, this is the first time such a formula has been analytically established to consider the gap size and experimentally validated. A more accurate multiphase description of the physical phenomenon could be developed by including fluid effects using CFD tools, such as possible lubrication between cells and the pillar walls.

## Figures and Tables

**Table 1:** Summary of morphological parameters of tumor cells.

Cell Type	Diameter (µm)	Stiffness (Pa)
A549	17 (this work)	430 [40]
MCF-7f	20 [51]	360 [40]
PC3-9	18 [52]	130 [52]
HeLa	15 [53]	387 [52]
MDA-MB-231	15.5 [52]	206 [52]
SW480	11 [53]	580 [54]

## Data Availability

The data that support the findings of this study are available within this article.
